# Hypergravity disrupts murine intestinal microbiota

**DOI:** 10.1038/s41598-019-45153-8

**Published:** 2019-06-28

**Authors:** Corentine Alauzet, Lisiane Cunat, Maxime Wack, Alain Lozniewski, Hélène Busby, Nelly Agrinier, Catherine Cailliez-Grimal, Jean-Pol Frippiat

**Affiliations:** 1 Université de Lorraine, SIMPA, F-54000 Nancy, France; 2 Laboratoire de Bactériologie, Centre Hospitalier Régional Universitaire Nancy, F-54000 Nancy, France; 3 CHRU-Nancy, INSERM, Université de Lorraine, CIC, Epidémiologie Clinique, F-54000 Nancy, France; 4 Département d’anatomie et cytologie pathologiques, Centre Hospitalier Régional Universitaire Nancy, F-54000 Nancy, France

**Keywords:** Microbiome, Medical research

## Abstract

During spaceflight, organisms are subjected to various physical stressors including modification of gravity (G) that, associated with lifestyle, could lead to impaired immunity, intestinal dysbiosis and thus potentially predispose astronauts to illness. Whether space travel affects microbiota homeostasis has not been thoroughly investigated. The aim of this study was to evaluate changes in intestinal microbiota and mucosa in a ground-based murine model consisting in a 21-days confinement of mice in a centrifuge running at 2 or 3G. Results revealed an increased α-diversity and a significant change in intracaecal β-diversity observed only at 3G, with profiles characterized by a decrease of the *Firmicutes*/*Bacteroidetes* ratio. Compared to 1G microbiota, 12.1% of the taxa were significantly impacted in 3G microbiota, most of them (78%) being enriched. This study shows a G-level-dependent disruption of intracaecal microbiota, without alteration of mucosal integrity. These first data reinforce those recently obtained with in-flight experimentations or microgravity models, and emphasize the critical need for further studies exploring the impact of spaceflight on intestinal microbiota in order to optimize long-term space travel conditions.

## Introduction

The intestinal microbiota is a complex microbial ecosystem whose balance and homeostasis are maintained by a constant influx of commensal microorganisms. The intestinal microbiome is a dynamic entity which was shaped by life events and has the ability to adapt itself in terms of diversity and community structure with an individual trajectory of assembly^1–3^. A healthy microbiome, even in a stable state, exhibits variations in space and time in the relative abundance of the autochthonous microorganisms indicating a temporally adaptive natural response in the host, leading to the concept of a « personal microbiome »^4^. The complexity and variation of the resilience of the microbiota between and within individuals make data exploitation difficult. Nevertheless, it is now recognized that the human intestinal microbiota is represented by two predominant phyla, *Firmicutes* and *Bacteroidetes*, and by some minor ones mainly represented by *Actinobacteria*, *Proteobacteria*, *Fusobacteria* and *Verrucomicrobia* members^1,2,5^. Most of these taxa play essential physiological functions such as breaking down fibers, stimulating the immune system and preventing pathogen colonization^6^. Microbiota composition is affected by both intrinsic factors like host genetics, and extrinsic factors like medication or diet^7–10^. An increasing number of studies have also shown that stress, particularly chronic stress, has profound effects on the composition and organization of the gut microbiota either directly by the action on bacteria of stress mediators released in the lumen, or indirectly by modulating local immunity, intestinal motility or visceral perception^11–14^.

Spaceflights represent a unique stress model dealing with consistent or intermittent stressors of psychosocial (confinement, isolation, sleep deprivation, persistent circadian misalignment) or physical origin (hypergravity generated during take-off and landing, microgravity present throughout the flight, solar and cosmic radiations)^15–17^. Using a monofactorial murine stress model based on long-term hypergravity, we previously showed that hypergravity increases serum corticosterone concentration and anxiety-like responses^18^. These modifications were dependent on the level of hypergravity since they were observed at 3G and 4G but not at 2G. In astronauts, despite anticipation and exceptional training to manage physical and mental stressors before spatial missions, activation of adrenocortical and glucocorticoid stress-response pathways have been observed during and after spaceflight^19^. Interestingly, sympathetic nervous system, as well as hypothalamic-pituitary-adrenal (HPA) axis, are also strongly involved in the brain-gut axis that drives communication between the central nervous system and the gastrointestinal tract, including microbiota^20^. Modulation of these communications could impact the stability and composition of the intestinal microbiota. Dysregulation of the immune system, that could also modulate gut microorganisms, was frequently described in animals and astronauts. Alteration of B and T lymphopoiesis were also described in ground-based murine model of hyper- or microgravity^16,18,21–23^. In astronauts, deregulation of cell-mediated immunity such as altered T cell memory subset distribution, altered cytokine production profiles or decreased delayed-type hypersensitivity, were described in association to compromised defenses against pathogenic microorganisms^16,19^. Numerous other factors encountered during spaceflight could influence gut bacteria such as nutritional factors or radiations^24^. Moreover, it has been shown that microorganism behavior is modified during spaceflights^15,17,25,26^. Depending on studied bacteria, enhanced or decreased virulence^15,25^, altered antimicrobial susceptibility^27,28^ and/or increased biofilm formation^15,28,29^ have been described, due to, for instance, a modulation of the regulation of gene expression^15,25,26,30,31^.

All these parameters could lead to direct or indirect alterations of intestinal communities during spaceflight. As imbalance in gut microbiota could be correlated with a shift from a healthy state to a diseased state, it is important to evaluate the status of the astronauts’ gut microbiomes for long-duration space missions and the role of each parameter in dysbiosis development^32^. The objective of this study was to evaluate changes in intestinal microbiota and mucosa in a ground-based murine model consisting in the confinement of mice during 21 days in a centrifuge running at 2 or 3G. These two levels of hypergravity were chosen to determine the potential impact of persistent psychosocial stress observed only at 3G^18^.

## Results

### Intestinal microbiota diversity is significantly altered in mice exposed to 3G-hypergravity

**Figure 1:**
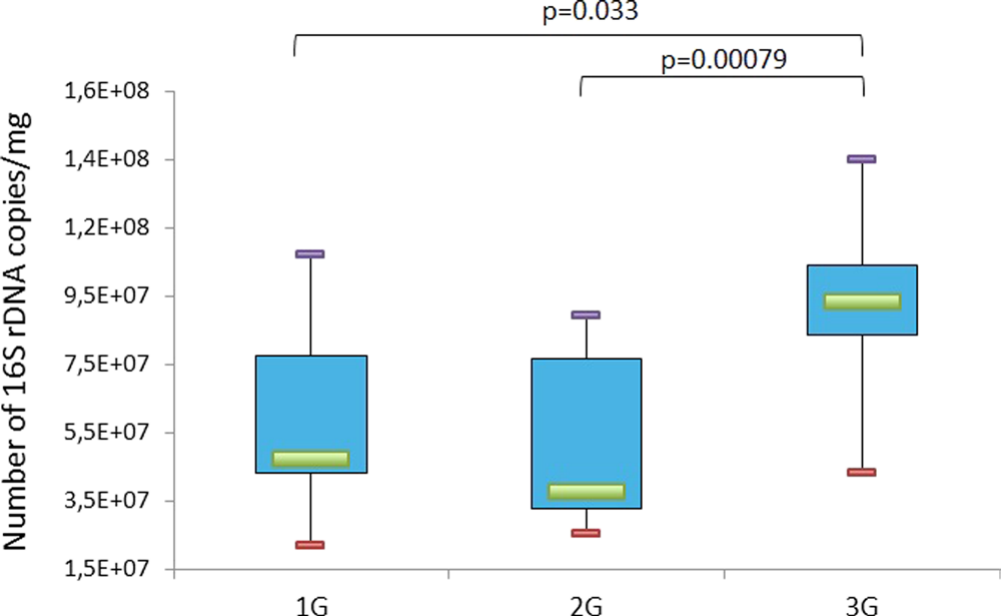
Total bacterial load quantification by qPCR corresponding to the total number of 16S rRNA encoding gene copies per mg of intracaecal content of mice subjected to 2G (n = 10) or 3G (n = 11) hypergravity and 1G control mice (n = 7). Statistical analyses were done using the Mann-Whitney U test. The upper and lower ranges of the box represent the 75% and 25% quartiles, respectively. Error bars reflect standard error of the mean.

Mice were divided in three groups: 12 mice centrifuged at 2G, 12 mice centrifuged at 3G, and 8 controls (1G) placed in the same experimental conditions but in a static position. All groups were exposed to these conditions during 21 days with 4 animals per cage. After 21 days, animals presenting injuries (such as bites that could induce inflammation) were discarded resulting in ten 2G mice, eleven 3G mice and seven controls (1G). Intracaecal content was collected for each of these mice (28 samples) and analyzed by pyrosequencing and qPCR (16S rDNA quantification) as described in the Materiel & Methods section. Quantification of intracaecal 16S rDNA amounts showed that bacterial load was significantly increased in mice subjected to 3G-hypergravity (Fig. 1). Our pyrosequencing protocol led us to generate an average of 14,476 reads per sample (ranging from 8,054 to 25,136) with a mean length of 525 bp (ranging from 498 to 533 bp). Individual rarefaction curves (Suppl. Fig. S1) showed that the mean number of observed operational taxonomic units (OTUs) is 155 (ranging from 110 to 195 OTUs), with all samples reaching a plateau at approximately 5,000 sequence reads. The read coverage was thus sufficient to capture most of the bacterial diversity of each intracaecal microbiome.

**Figure 2:**
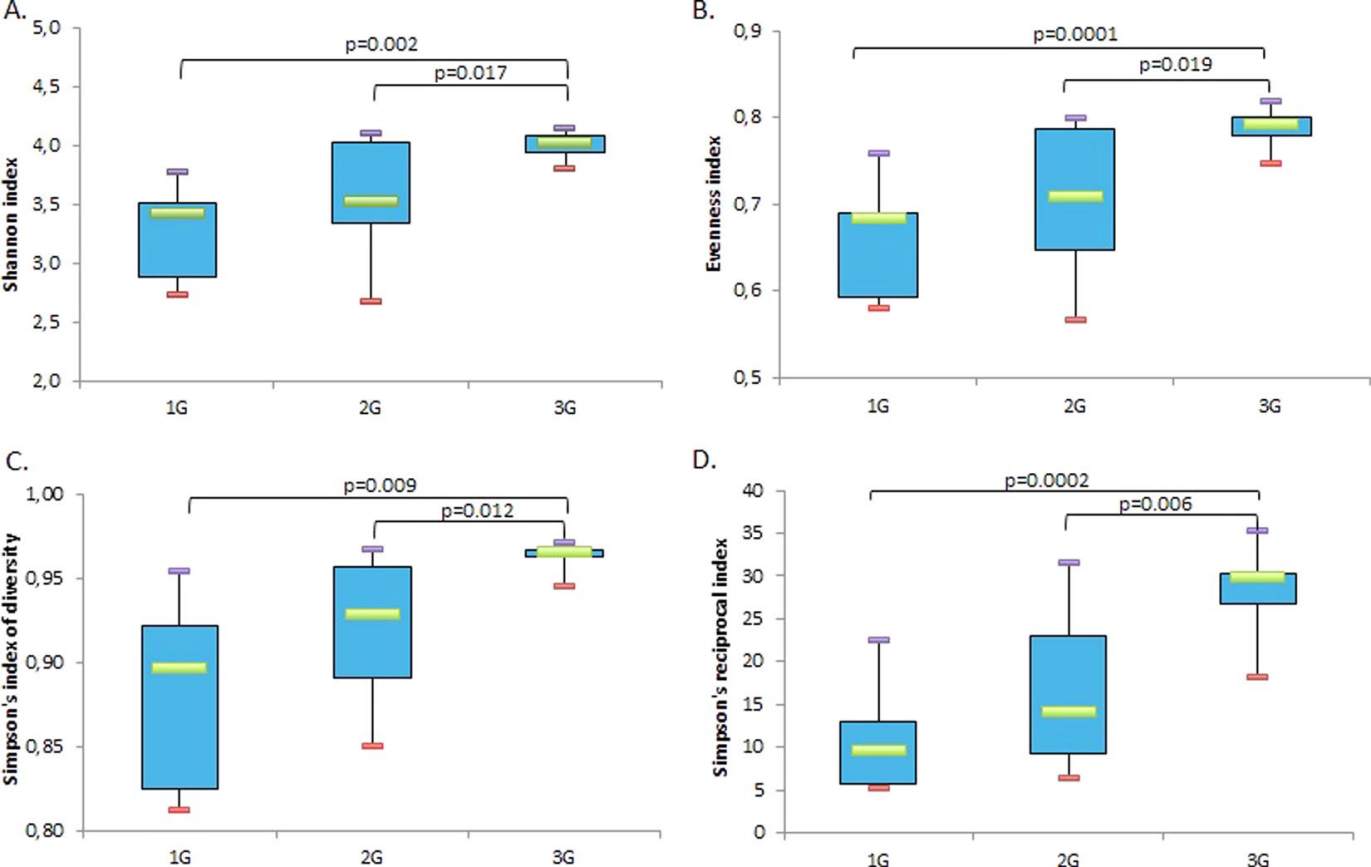
Phylogenetic diversity of murine intracaecal microbiomes at 1G (*n* = 7), 2G (*n* = 10) or 3G (*n* = 11). Box plots depict microbiomes diversity differences according to the (**A**) Shannon index, (**B**) Evenness index, (**C**) Simpson’s diversity index and (**D**) Simpson’s reciprocal index. Statistical analyses were done using the Mann-Whitney U test. The upper and lower ranges of the box represent the 75% and 25% quartiles, respectively. Error bars reflect standard error of the mean.

**Figure 3:**
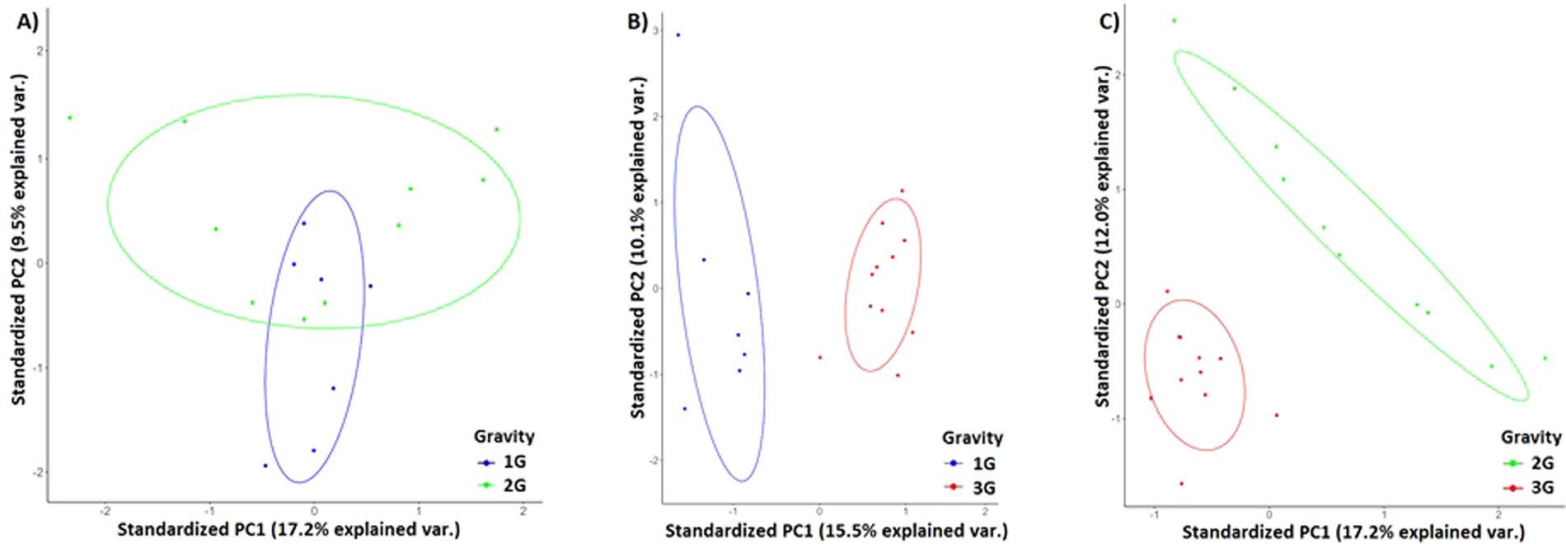
Principal Component Analysis (PCA) of microbiomes of (**A**) 2G mice versus 1G mice; (**B**) 3G mice versus 1G mice, and (**C**) 2G mice versus 3G mice. The variance explained by each of the main two dimensions of the PCA is indicated in parentheses on the axes.

The greatest mean diversity was found in 3G-samples. A significant increase of the α-diversity was observed with all indexes used in the microbiota of mice subjected to 3G-hypergravity as compared to the microbiota of mice centrifuged at 2G and 1G (Fig. 2). Similarities between intracaecal communities assessed by principal component analysis (PCA) did not show real demarcations between 1G and 2G microbiota while a clear clustering between samples from 1G and 3G microbiota as well as from 2G and 3G microbiota was observed (Fig. 3).

### Intestinal microbiota composition is modified by hypergravity

A more in-depth taxonomic analysis of bacterial types led us to highlight taxa influenced by hypergravity (Table 1, details in Suppl. Database S1). Only few variations occurred in microbiota of mice submitted to 2G-hypergravity in comparison to 1G mice, whatever the phylogenetic level observed. These variations were clearly greater in microbiota of 3G mice and occurred at all phylogenetic levels.

**Table 1:** Number of OTUs impacted (p < 0.05 or * = 0.05 < p < 0.1) by the level of hypergravity in their relative abundance (RA) according to the taxonomic level (for details, see Suppl. Database S1).

Taxonomic range	Total No of OTUs	No of OTUs that significantly differed in their RA from:								
		2G *vs* 1G			3G *vs* 1G			3G *vs* 2G		
		Total	increase [emergence]	decrease [extinction]	Total	increase [emergence]	decrease [extinction]	Total	increase [emergence]	decrease [extinction]
Phylum	7	0	—	—	1	—	1 *Proteobacteria*	2	—	2 *Proteobacteria* *Verrucomicrobia*
Class	18	0	—	—	2		1 + [1] *Betaproteobacteria* [*Cytophagia*]	2	—	2 *Coriobacteriia Verrucomicrobiae*
Order	23	0	—	—	2	—	1 + [1] *Burkholderiales* [*Cytophagales*]	2	—	2 *Eggerthellales Verrucomicrobiales*
Family	51	3	1 *Rikenellaceae*	1 + [1] *Hymeno-bacteraceae* [Cytophagaceae*]	11	6 + [2] *Bacillaceae* *Caldicoprobacteraceae* *Enterococcaceae* *Lachnospiraceae* *Rikenellaceae* *Ruminococcaceae* [unclassified Bacteroidales] [Carnobacteriaceae*]	1 + [2] *Sutterellaceae* [*Cytophagaceae*] [*Hymeno-bacteraceae*]	12	7 + [1] *Bacillaceae Clostridiaceae* *Enterococcaceae* *Odoribacteraceae* *Oscillospiraceae* *Prevotellaceae* *Ruminococcaceae* [*Carnobacte-riaceae*]	4 *Akkermansiaceae Eggerthellaceae* *Streptococcaceae* *Sutterellaceae*
Genus	118	3	2 *Alistipes* *Anaerocolumna*	1 Pontibacter*	13	7 + [1] *Alistipes* *Anaerocolumna* *Caldicoprobacter* *Enterococcus* *Marvinbryantia* *Roseburia* *Ruminococcus* [*Syntrophococcus*]	4 + [1] Allobaculum* Catenisphaera* Enterorhabdus* *Parasutterella* [*Pontibacter*]	11	7 *Enterococcus* *Hungatella* *Marvinbryantia* *Odoribacter* *Ruminococcus* *Syntrophococcus* *Turicibacter*	4 *Akkermansia* Catenisphaera* *Enterorhabdus* *Parasutterella*

**Figure 4:**
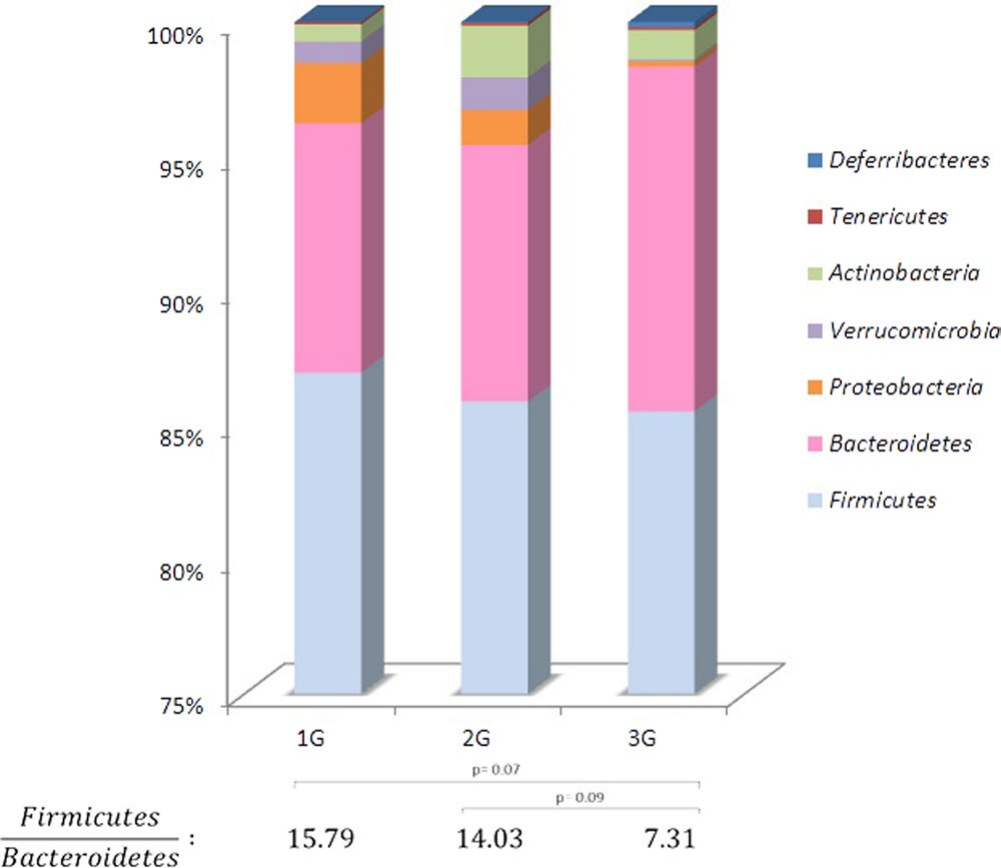
Relative abundance (%) of bacterial phyla and *Firmicutes/Bacteroidetes* ratio in the caecal content of mice subjected to hypergravity at 2G and 3G compared to control mice (1G). A significant decrease of *Verrucomicrobia* (p = 0.0095) was noted in 3G samples by comparison to 2G samples. Significant lower levels of *Proteobacteria* (p = 0.0224 and 0.0095) were noted in 3G samples by comparison to 1G and to 2G samples (p = 0.0224 and p = 0.0095, respectively). Furthermore, higher levels of *Deferribacteres* (p = 0.078) were noted in 3G samples by comparison to 1G samples. Statistical analyses were done using the Mann-Whitney U test.

The caecal microbiota consisted of bacteria from 7 divisions. Most of the identified sequences belonged to the *Firmicutes* (ranging from 71.8 to 93.3%) and *Bacteroidetes* (ranging from 2.2 to 26.5%) phyla. In 3G mice, we observed higher levels of *Bacteroidetes* by comparison to 1G and 2G mice, associated to a significant decrease of the *Firmicutes*/*Bacteroidetes* ratio (Fig. 4). Among the other phyla, we observed a significant decrease at 3G in the relative abundance of *Proteobacteria* members (p = 0.0224 by comparison to 1G; p = 0.0095 by comparison to 2G) and an increase of *Verrucomicrobia* members at 3G by comparison to 1G microbiota (p = 0.0095). A seventh phylum, *Deferribacteres*, appeared in microbiota of mice subjected to hypergravity with an increase more noticeable at 3G (p = 0.078 by comparison to 1G).

At the class and order taxonomic levels, the few significant changes observed have concerned only lowering of taxa at 3G (Table 1).

Among the 53 identified families (Table 1), only 3 were significantly impacted at both levels of hypergravity (1 enhanced, 1 decreased and 1 disappearing), 8 differed in 3G microbiomes compared to 1G (7 enhanced and 1 decreased), and 7 other families were only impacted at 3G compared to 2G (4 enhanced and 3 decreased).

Of the 118 genera recovered (Table 1), two of them were significantly increased at both levels of hypergravity (*Anaerocolumna* and *Alistipes*) whereas an impoverishment of a unique genus, *Pontibacter*, was noted. Of the 14 genera whose relative abundance was modified only at 3G, some of them were dominant members of mice microbiota. Indeed, although not statistically significant, a 16-fold decrease of *Catenisphaera* (from 8.40% to 0.52%; p = 0.079) and an 11-fold decrease of *Allobaculum* (from 4.07% to 0.38%; p = 0.054) were observed. Moreover, a 3-fold increase of *Ruminococcus* (from 2.53% to 7.46%; p = 0.020) and a 14-fold increase of *Marvinbryantia* (from 0.48% to 6.78%; p = 0.054) were noted.

**Figure 5:**
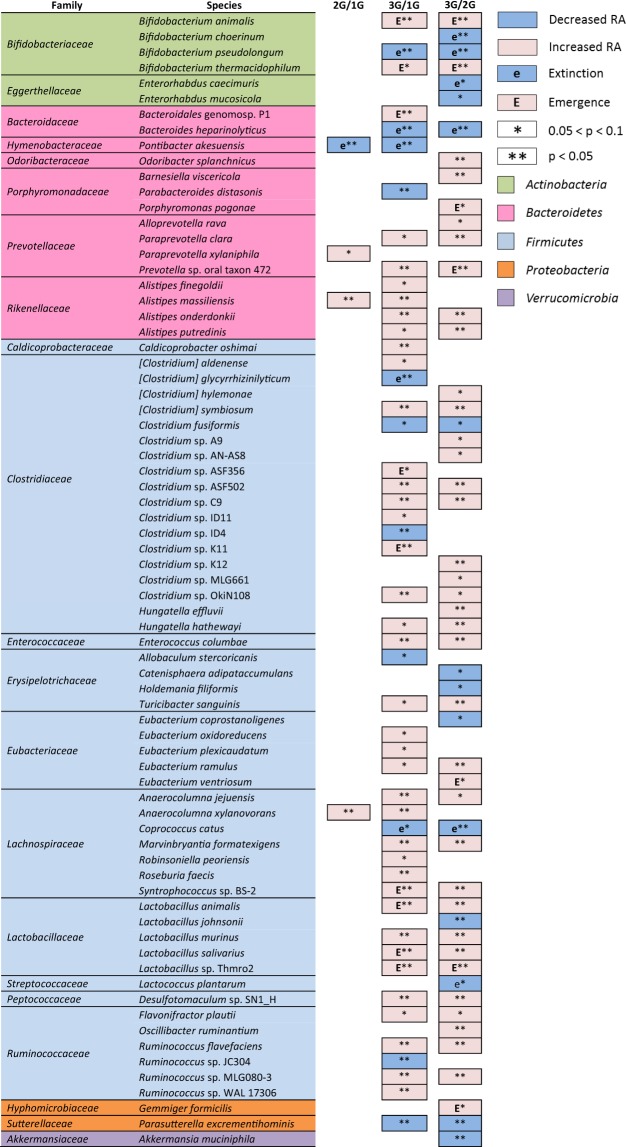
Bacterial species significantly enriched or impoverished in 2G samples compared to 1G samples (2G/1G), and in 3G samples compared to 1G samples (3G/1G) and to 2G samples (3G/2G). Taxa names in brackets correspond to species whose classification has to be re-evaluated. Statistical analyses were done using the Mann-Whitney U test.

**Figure 6:**
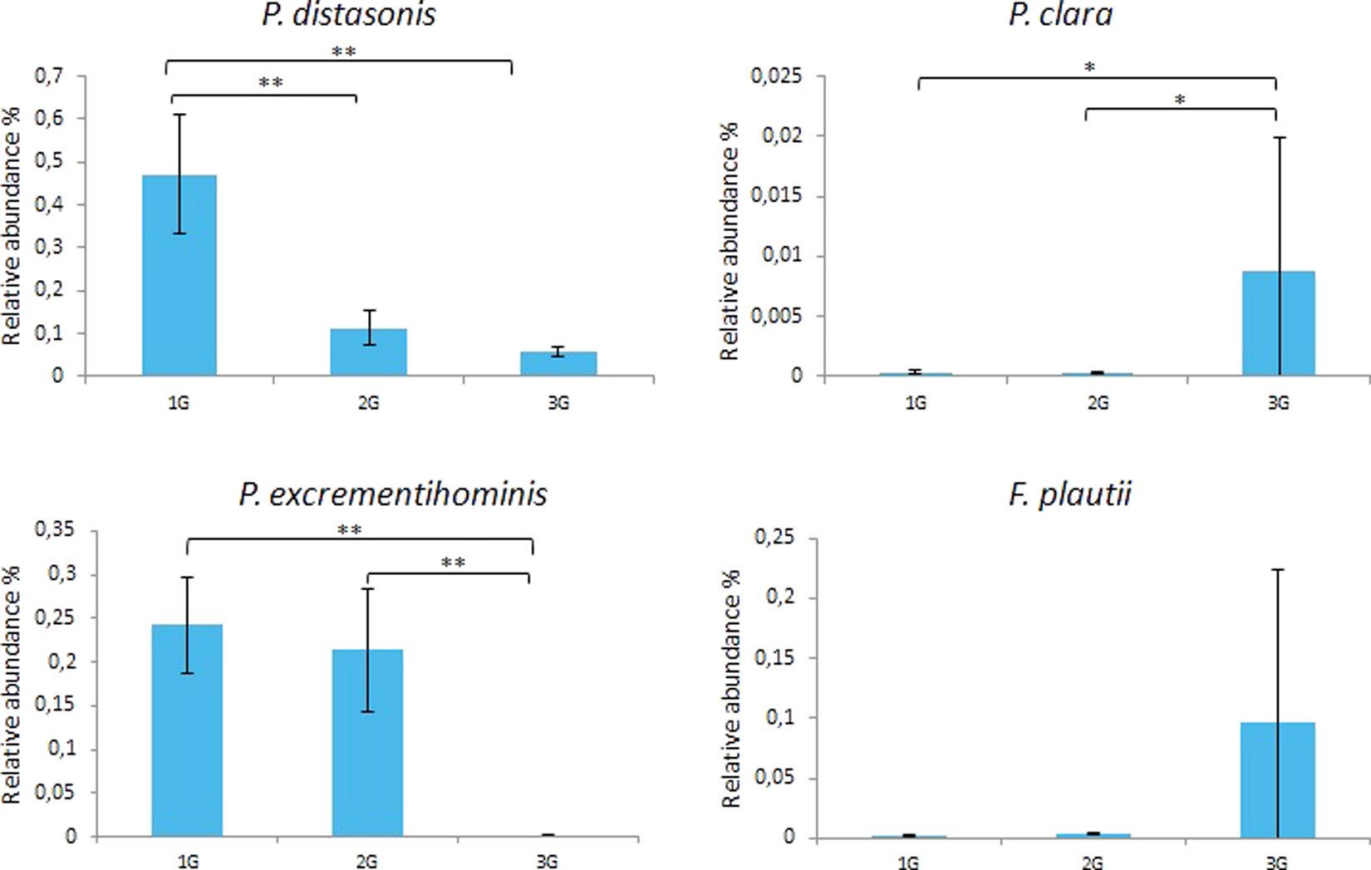
Intracaecal 16S rDNA gene-based real-time PCR quantification of the relative abundance of *Parabacteroides distasonis*, *Paraprevotella clara*, *Parasutterella excrementihominis*, and *Flavonifractor plautii*. Data are expressed as the relative proportion of the number of 16S rDNA copies of each target to the total number of 16S rDNA copies per mg of intracaecal content of mice subjected to 2G (*n* = 10) or 3G (*n* = 11) hypergravity and 1G control mice (*n* = 7). Statistical analyses were done using the Mann-Whitney U test. *p < 0.05; **p < 0.0005. Error bars reflect standard error of the mean.

At the species level, 414 taxa were assigned, 20 of them representing around 70% of the bacteria load. Only 28 species were recovered from all animal groups (Suppl. Fig. S2). The most abundant species in all samples was *Faecalitalea cylindroides*, a member of the *Erysipelotrichaceae* family (*Firmicutes*), averaging 20.7% of the microbiota in the 1G mice, 16.7% in the 2G mice and 5.7% in the 3G mice. Despite this decrease, statistical analysis showed that these taxa’s impoverishment were not significant due to too much intra-group variations. Bacterial species significantly enriched or impoverished are presented in Fig. 5 (Suppl. Database S1, Suppl. Fig. S3). Compared to 1G microbiota, only 1% of the species presented a relative abundance that was significantly impacted at 2G whereas 12.1% of the species presented a relative abundance significantly altered at 3G hypergravity. Of the 50 species significantly impacted at 3G-hypergravity level, 78.0% were enriched compared to 1G microbiota with relative abundance that could be strongly enhanced (for example, *Alistipes onderdonkii*, 63-fold; *Lactobacillus murinus*, 51-fold; *Prevotella* sp. oral taxon 472, 30-fold; *Enterococcus columbae*, 22-fold; *Marvinbryantia formatexigens*, 14-fold). Two taxa were enriched at both level of hypergravity: *Alistipes massiliensis* (28-fold at 2G; 11-fold at 3G) and *Anaerocolumna xylanovorans* (5-fold at 2G; 4-fold at 3G). Hypergravity at 3G also led to a strong impoverishment in relative abundance of 22% (11 of 50 taxa when compared to 1G) and 26.9% (14 of 52 taxa when compared to 2G) of the intracaecal species significantly impacted. Among the species specifically decreased in 3G samples, some taxa represented major members of the 1G microbiota such as *Catenisphaera adipataccumulans* (8.8%, 16-fold decrease), *Clostridium* sp. ID4 (6.7%, 10-fold decrease), *Clostridium populeti* (1.8%, 7-fold decrease) and *Lactobacillus johnsonii* (1.5%, 3-fold decrease). *Pontibacter akesuensis* was the only species that was decreased at both levels of hypergravity. qPCR specifically targeting four taxa of interest that were significantly impacted by hypergravity confirmed pyrosequencing results (Fig. 6).

### 2G and 3G hypergravity did not affect intestinal structure and local immunity

Histological examination of intestinal tissues did not reveal differences between colonic and caecal tissues obtained from centrifuged and 1G animals. Indeed, in all animals, we observed the absence of inflammatory lesion or neutrophil infiltration as well as the absence of submucosal plexitis (Suppl. Fig. S4). The Riley score ranged from 0 to 1 in 1G, 2G and 3G mice. Examination of villous height and crypt depth did not either reveal variation of intestinal integrity in mice subjected to hypergravity. In order to correlate these observations with local immunity, we quantified in caecal tissues the mRNA levels of two immunological markers, IgM heavy chains and RORγ (a marker of Th17 cells and ILC3s, a subtype of innate lymphoid cells that confer protection at the mucosal frontier), and of the stress marker NR3C1 (glucocorticoid receptor). Results did not either reveal significant differences in their mRNA expression in caecal tissues (Suppl. Fig. S5).

## Discussion

There is growing evidence indicating the impact of stress on intestinal homeostasis and that alterations in microbiome composition can lead to local or central dysregulations that could be involved in the onset or exacerbation of chronic disorders such as intestinal bowel diseases (IBD)^33^. During spaceflight, astronauts are subjected to various physical and psychosocial stressors, including modification of gravity, which could lead to dysbiosis. Whether space travel affects intestinal equilibrium has not been thoroughly investigated particularly due to constraints imposed by in-flight experimentation^24^. To overcome these limitations, ground-based devices simulating microgravity have been used to explore intestinal diversity in mice but no study has yet analyzed the impact of hypergravity^31,34^. In this study, we analyzed and compared the intracaecal communities of mice subjected to 2 levels of hypergravity and control mice.

Results revealed that hypergravity influenced intestinal microbiota composition and that its impact was clearly more important at 3G than at 2G. Increased bacterial load and α-diversity as well as significant change in intracaecal global β-diversity were observed only at the higher level of hypergravity. These results could be related to previous works which demonstrated that only 3G-hypergravity induced durable HPA axis activation associated with enhanced serum corticosterone level and persistent anxiety behaviors 15 days after returning to normogravity^18^. In this previous work, we also observed a decrease in plasmatic chemokines and Th1 cytokines after 3G centrifugation^18^. So, as for stress pathway activation or immune system modulation, the consequences of gravity on intestinal microbiota seem to be G-level dependent. Consequently, we can hypothesize that the effects are mainly due to psychosocial stress persisting only at 3G. More and more studies are exploring the influence of stress on intestinal microbiota^11,12,35–38^. Some of them described results similar to ours with, for example, a decreased *Firmicutes*/*Bacteroidetes* ratio^12,39^ but others observed either opposite effects or no impact^11,36,38^. Such variability could be due to factors such as the type of stress model, the origin of the samples (fecal, intraluminal or mucosal) or protocol parameters (DNA extraction method, PCR parameters)^40,41^. For example, by using the same murine model of stress (female C57/BL6 mice submitted to water-avoidance stress), Sun *et al*.^38^ described after pyrosequencing an increased intestinal bacterial load, concordant with our results, whereas Aguilera *et al*.^35^ observed no effect of stress on total bacterial count determined by FISH. Whatever the model used, the majority of the studies described an impact of stress on β-diversity and so, on intestinal communities. It is noteworthy that only few bacterial taxa were shared by all animals, stressed or not, suggesting the existence of only a reduced core microbiome. The absence in intracaecal samples of some species frequently recovered from murine gut such as SFB or the newly described ‘*Muribaculaceae’* (formerly S24-7 group) is also surprising^12,42,43^. Such discrepancies could be explained by the tremendous diversity of gut microbiota that could be due to differences in mice strain, age, gender or alimentation.

At lower taxonomic levels, we observed various effects of hypergravity. Although the relative abundance of *Firmicutes* was not impacted by hypergravity, an increased proportion of *Clostridia* and *Clostridiales* was observed at 3G, linked notably with the enhancement of some major members of the microbiota that are present in both humans and mice gut. These results are in accordance with in-flight experimentation^24^ or microgravity model^31^, as well as other models of stress^11^ or IBD^44^ that have described a burst of *Clostridiales* in mice gut. *Clostridia* represent a catch-all classis that contains a lot of beneficial taxa such as *Lachnospiraceae* or *Ruminococcaceae* members^39,45^ but also some deleterious members such as *Flavonifractor plautii* which has the capacity of cleaving protective compounds such as the antioxydative flavonoid quercetin^44,46^. Moreover, it has been shown that a cocktail of several *Clostridiales* has an impact on the generation of free intraluminal catecholamine in the gut through by their β-glucuronidase activity, and thus, could interfere with stress response^47^. Among *Firmicutes*, we also noticed no change in *Lactobacillales* abundance. Nevertheless, at the species level, some protective taxa decreased (*L. johnsonii*) while other increased (*L. murinus*) potentially offsetting each other.

For the *Bacteroidetes* phylum, although not significant, an increase was noticed at 3G which is in agreement with results of Ritchie *et al*.^24^ obtained in mice subjected to a 13-day spaceflight as well as results obtained using other stress models^12,39^. Rare taxa were increased at both levels of hypergravity, some of them being potentially deleterious such as members of the *Paraprevotella* genus that have been described as being more prevalent in intestinal lumen of patients with colorectal cancer^48^. However, it is interesting to note that only few taxa were decreased at both levels of hypergravity: *P. akesuensis* that disappeared and *Parabacteroides distasonis* that clearly declined. This latter taxon, recovered from human and mice gut, seems to have beneficial effects. Indeed, its relative abundance was significantly decreased in a mouse model of social disturbance stress^49^ as well as in inflammatory biopsies of patients with IBD^50^. Moreover, Kverka *et al*.^51^ showed that the administration of this species attenuated colitis in mice. It is therefore likely that this species, although a minority in gut microbiota, plays a protective role, highlighting the impact of its stress-related loss.

Finally, among other intestinal phyla, a significant diminution of *Proteobacteria* was observed at 3G unlike results obtained in other stress models or IBD^38,52^. Surprisingly, we observed an important decrease of *Parasutterella excrementihominis*, a species that has been correlated with inflammatory processes^44,53^.

Concerning the impact of gravity modification on intestinal tissues, limited reports have suggested that spaceflight or terrestrial models of microgravity affect the integrity of intestinal epithelium in rats^31,34^. Li *et al*.^34^ reported tissue damages and immune cell infiltration in colon sections of mice submitted to simulated microgravity. As reported by Ritchie *et al*.^24^ for mice submitted to short spaceflight, we observed no significant morphological alterations of the intestinal epithelium. In other models of chronic stress, alteration of intestinal epithelium was variably observed and mostly absent or moderate^36^.

In agreement with histological studies, no significant difference was observed in local expression of immunological markers. Similar results were observed in another murine model of stress, prolonged restraint stress, which had only little effects on mucosal immunity with unaffected colonic IgA levels and cytokine gene expression^36^. However, our results should be further confirmed by using a flow cytometry approach that will permit to accurately quantify all immune populations recovered within the gut. Moreover, although it has been shown that locally produced glucocorticoids can impact immunity^54^, the glucocorticoid receptor NR3C1 was not expressed differently in hypergravity and control mice, corroborating the absence or low impact of hypergravity on stress-induced mucosal immunomudulation. The absence of local increase of glucocorticoid receptors even at 3G despite increased serum corticosterone concentration^18^ is in agreement with observations obtained in thymuses of mice pups conceived and born at 2G^21^.

## Conclusion and Perspectives

Our work explored for the first time the impact of hypergravity on intracaecal microbiota and revealed modified bacterial communities, particularly at 3G, without alteration of mucosal integrity. Altogether, our results are similar to data obtained with in-flight experimentation^24^, indicating that hypergravity obtained by centrifugation is a useful ground-based murine model. Furthermore, the existence of a deep link between intestinal microbiota and immune system highlights the need for studies improving our knowledge about the impact of spaceflight on both systems in order to optimize long-term space travel conditions. Indeed, dysbiosis that may appear during spaceflight could have an impact not only on immune system efficiency, but also on energy intake, nutriments assimilation and intermediary metabolisms such as those of antibiotics^55,56^. It is only recently that these critical issues have begun to receive the attention they deserve. Moreover, among bacterial taxa impacted by hypergravity, some of them should be closely investigated to explore their deleterious or beneficial effects within the gut. Data obtained from such studies are important as they could lead to new strategies, such as pre- or probiotics supplementation or dietary approaches, to countermeasure spaceflight-associated dysbiosis and its consequences on health.

## Methods

### Animals

Experimental procedures were carried out in conformity with the National Legislation and the Council Directive of the European Communities on the Protection of Animals Used for Experimental and Other Scientific Purposes (2010/63/UE). Experiments were approved by the French Ministry of Research (authorization 04827) and the local ethic committee (Comité d’Ethique Lorrain en Matière d’Expérimentation Animale, agreement number: CELMEA-2012-0008). Mice used in this study were C57BL/6j males (8-week-old, mean body mass of 20 g) purchased from Charles River (Les Oncins, France). For acclimation to room conditions, they were housed during a week in standard cages (four mice per cage, 36 × 20 × 14 cm) in a quiet room under constant conditions (22 °C, 50% relative humidity, 12-h light/dark cycles) with free access to standard food and water.

### Centrifugation

As described previously^18^, standard cages containing mice were placed in the gondolas of a large-radius centrifuge with a rotational speed producing a gravity of 2G or 3G. Cages containing control mice were placed in the same gondolas and in the same room as centrifuged mice but in a static position (1G), so that all environmental variables, except the gravity level, were the same. Mice were supplied enough food and water for three weeks, so that the centrifuge was operating continuously. Mice were left undisturbed during the three weeks of centrifugation. Infrared video cameras fixed on centrifuge arms allowed remote day and night monitoring of mice. At day 21, the centrifuge was stopped and experimental groups (1G, 2G and 3G) were anesthetized using isoflurane, weighed and then euthanized by cervical dislocation. All samples were immediately processed to avoid degradation and/or contamination.

### Sample collection

The intestine was dissected in by excising the entire caecum and the most proximal part of the colon. Samples were opened longitudinally and their contents were removed by two successive washes in DEPC (1‰)-treated PBS. Intra-luminal contents were immediately frozen in liquid nitrogen and stored at −80 °C until bacterial DNA isolation. Five-millimeter-long specimens of tissue from each sample were fixed in 4% neutral-buffered formalin for histological analysis. Remaining tissues were placed in 1 ml of Trizol, frozen in liquid nitrogen and stored at −80 °C until RNA isolation.

### Histological analysis

Specimens were dehydrated and embedded in paraffin. Five µm paraffin sections were stained with HES (hematoxylin, eosin, saffron). In total, 56 caecal and colonic biopsies were examined. For each biopsy specimen, nine histological features were assessed. The Riley score which evaluate the acute inflammatory cell infiltrate, crypt abscesses, mucin depletion, ulceration, chronic inflammatory cell infiltrate and crypt architectural irregularities was calculated^57^. Plexitis, mucosa height and intraepithelial lymphocytosis were also appraised^58^. Inflammatory cell, intraepithelial lymphocytosis, mucin depletion and plexitis were graded on a four point scale corresponding to none, mild, moderate and severe. Plexitis was defined by the presence of one or several inflammatory cells (lymphocytes, plasmocytes, eosinophils, mast cells) in submucosal plexus. Crypt architectural irregularities and ulceration were counted with a binary scale, 0 for none or 1 for any lesion. Evaluation of villi height was performed using a Vision Tek Sakura Live Digital Microscope. The others items were evaluated in light microscopy, using an Olympus BX60 Microscope.

### DNA isolation

Whole genomic DNA was extracted from caecal samples (50 mg) using the Fast DNA SPIN kit for Soil (MP Biomedicals, Santa Ana, CA, USA) after bead beating with the FastPrep-24 Instrument (MP Biomedicals) at 6.0 ms^−1^ for 40 s, according to manufacturer’ s instructions. This method was chosen following a preliminary study comparing the efficiencies of seven widely used methods (6 commercial and 1 manual) for DNA isolation from mice feces and caecal contents^40^. Purified DNA was resuspended in sterile deionized Dnase/pyrogen-free water, analyzed by spectrophotometry (NanoDrop 2000C; Labtech, Heathfield, East Sussex), and frozen (−20 °C) for further analysis (described below).

### Fecal microbiota sequencing

Initial amplification of the V4-V6 region of the 16S rRNA gene was performed using barcoded primers Bact-515F (5′-GTGCCAGCMGCNGCGC-3′) and Bact-1061R (5′-CRRCACGAGCTGACGAC-3′) described by Klindworth *et al*.^59^ in which the barcode was an 11-bp sequence unique to each sample. PCR reactions contained 2.5 U of *Taq* DNA Polymerase (Invitrogen, Cergy Pontoise, France), 5 µl of 5X buffer, 75 nmol MgCl_2_, 1 µl of 10 mM dNTPs, 1 µl of each primer (50 µM) and 50 ng of DNA. Three PCR reactions were run for each sample as follows: 95 °C for 5 min, followed by 40 cycles at 95 °C for 45 s, 60 °C for 45 s, 72 °C for 45 s and a final extension at 72 °C for 5 min. PCR reactions from the same sample were pooled, purified using the QIAquick PCR purification kit (Qiagen, Courtaboeuf, France) and quantified using a Qubit 2.0 Fluorometer (Invitrogen) using the dsDNA HS Assay Kit. To ensure equal representation of each sample in the sequencing run, each barcoded sample was standardized by calculating equimolar amounts (100 ng/sample) using the SequalPrep Normalization Plate Kit (Invitrogen) prior to pooling. Pooled samples of the 16S rDNA multiplexed amplicons were sequenced on a Roche 454 Genome Sequencer FLX Titanium instrument using the GS FLX Titanium XLR70 sequencing reagents and protocols (Beckman Coulter Genomics, Danvers, USA).

### Amplicon sequencing data analysis

Analysis of amplicon sequencing data was carried out using the MEGAN pipeline^60^. After demultiplexing, combined raw sequencing data plus metadata were filtered to exclude low-quality reads. Next, data were denoised and clustered using the MIRA 4 software (http://mira-assembler.sourceforge.net). Sequences with ≥98% similarity were binned and assigned to the same OTU to approximate species-level phylotypes. Representative sequences of each OTU, derived from clusters or singletons, were assigned at different taxonomic level by using the Ribosomal Database Project II Classifier^61^. To avoid a potential bias linked to variation of sequence coverage between samples, the data were normalized to 100000 sequences per samples. Rarefaction curves were constructed to evaluate sequencing depth. Relative abundances of each OTU were compared according to the different experimental conditions. Bacterial richness and diversity across samples were estimated by calculating the following indexes: Shannon index (H′ = −∑ p_i_ ln(p_i_), where p_i_ equals the frequency of species i in the population), Evenness index (J  = $\frac{{\rm{H}}^{\prime} }{{\rm{Hmax}}}$ where Hmax equals the natural logarithm of total OTU’s number), Simpson’s diversity index ($D=1-\frac{{\sum }^{}{\rm{n}}({\rm{n}}-1)}{{\rm{N}}({\rm{N}}-1)}$ with N equals the total number of reads and n equals the number of reads of a particular species) and Simpson’s reciprocal index $(\frac{1}{{\rm{D}}})$. Principal Component Analysis (PCA) was conducted to appreciate overall distance between microbial communities, using relative abundance and taxa-to-taxa distance estimates. Obtained 16S rRNA gene sequences have been deposited into NCBI’s Sequence Read Archive (SRA) database under accession number SRP153133.

### Analysis of intracaecal bacterial load and quantification of specific OTU relative abundances by qPCR

Total bacteria amount was assessed by amplifying 0.5 ng of each fecal extract with pan-bacterial primers targeting the 16S rRNA gene. Specific qPCR targeting four taxa of interest (*Parabacteroides distasonis, Paraprevotella clara, Parasutterella excrementihominis* and *Flavonifractor plautii*) were developed. All PCR assays were performed using specific primers described in Supplementary Table S1 and MESA FAST qPCR MasterMix for SYBRAssay as recommended by the manufacturer (Eurogentec, Seraing, Belgium). Primers have been selected in the literature or created after *in silico* validation of their specificity and sensitivity for each targeted group by using the arb-SILVA online tool TestPrime (http://www.arb-silva.de) and the Ribosomal Database Project Probe Match pipeline (RDP II, http://rdp.cme.msu.edu). The standard curves used for quantification were optimized and based on serial dilutions of numerous bacterial DNAs extracted by using the QIAamp DNA Mini Kit (Qiagen). DNAs were extracted from strain *Barnesiella intestinihominis* DSM 21032^T^ for pan-bacterial qPCR, and from strains *P. distasonis* DSM 20701^T^, *P. clara* DSM 19731^T^, *P. excrementihominis* DSM 21040^T^ and *F. plautii* DSM 4000^T^ for specific qPCR. All assays were performed in triplicate. The following thermocycling conditions were applied with the MyiQ™2 real-time PCR system (Bio-Rad Laboratories): initial denaturation at 95 °C for 5 min followed by 40 cycles of 95 °C for 15 s and 60 °C for 1 min. Melting curves were obtained immediately after the amplification under the following conditions: 70 cycles of 10 s with an increment of 0.5 °C/cycle starting at 60 °C.

### Quantification by RT-qPCR of immunological and stress markers in intestinal tissues

To deepen our results, new sets of mice (n = 12 at 1G, n = 8 at 2G and n = 12 at 3G) were submitted to hypergravity using the same protocol as described above. After trizol elimination, total RNA was extracted from caecal tissues using the RNeasy kit (Qiagen) and reverse transcribed using random primers, RNAout and MML-V reverse transcriptase (Invitrogen) following manufacturer’s instructions. RT-qPCR were performed using the MESA Fast SYBRGreen I qPCR Master Mix (Eurogentec) and a Mastercycler realplex2 real-time PCR machine (Eppendorf, Hamburg, Germany). The cycling protocol was as follows: 3 min at 95 °C, followed by 40 cycles of 15 s at 95 °C and 30 s at the annealing temperature indicated in Supplementary Table S2. Each qPCR was performed in duplicate and repeated at least two times. Data analysis and relative expressions by comparison to 4 housekeeping transcripts (Eef2, Eif3f, Ppia, and HRPT1) were performed as previously described^62^. Amplification and quantification of immunological markers or stress markers encoding mRNA (IgM, Ror-γ, NR3C1) were done as previously described^21^. All primers used were targeted to different exons to ensure that they could not hybridize to potential traces of genomic DNA. Their specificity was checked using a BLAST search through the U.S. National Center for Biotechnology Information (Bethesda, MD, USA).

### Statistical analysis

Comparison of bacterial load quantification by qPCR, relative abundances, and phylogenetic diversity indexes were analyzed using the Mann-Whitney U test with a significance level α of 0.05. The p-values were adjusted for multiple hypotheses testing using the False Discovery Rate method^63^ for all the results within each taxonomy level. The PCA were produced using the ‘species’ taxonomic data. All the analysis were performed using R version 3.5.0 (https://www.R-project.org/).

## Supplementary information

Supplementary informations

Dataset

## Data Availability

All data generated or analyzed during this study are included in this published article (and its Supplementary Information files). 16S rRNA gene sequences are available into NCBI’s Sequence Read Archive (SRA) database under accession number SRP153133.
