# Supplementary material for: Hypergravity disrupts murine intestinal microbiota
Source: Sci Rep. 2019 Jun 28;9:9410. doi: 10.1038/s41598-019-45153-8 (PMC6599200; doi:10.1038/s41598-019-45153-8)
Supplement: Supplementary file 1 — Supplementary informations [file 41598_2019_45153_MOESM1_ESM.pdf]

Supplementary Information for

Hypergravity disrupts murine intestinal microbiota

Corentine Alauzet[1,2*], Lisiane Cunat[1], Maxime Wack[3], Alain Lozniewski[1,2], Hélène Busby[4], Nelly Agrinier[3], Catherine Cailliez-Grimal[1,#], Jean-Pol Frippiat[1, #].

[1] Université de Lorraine, SIMPA, F-54000 Nancy, France.
[2] Laboratoire de Bactériologie, Centre Hospitalier Régional Universitaire Nancy, F-54000 Nancy, France.
[3] CHRU-Nancy, INSERM, Université de Lorraine, CIC, Epidémiologie Clinique, F-54000 Nancy, France
[4] Département d'anatomie et cytologie pathologiques, Centre Hospitalier Régional Universitaire Nancy, F-54000 Nancy, France

*Correspondence and requests for materials should be addressed to C.A. (email: corentine.alauzet@univ-lorraine.fr)
[#]These authors contributed equally to this work.

Supplementary Figures

Supplementary Figure S1.

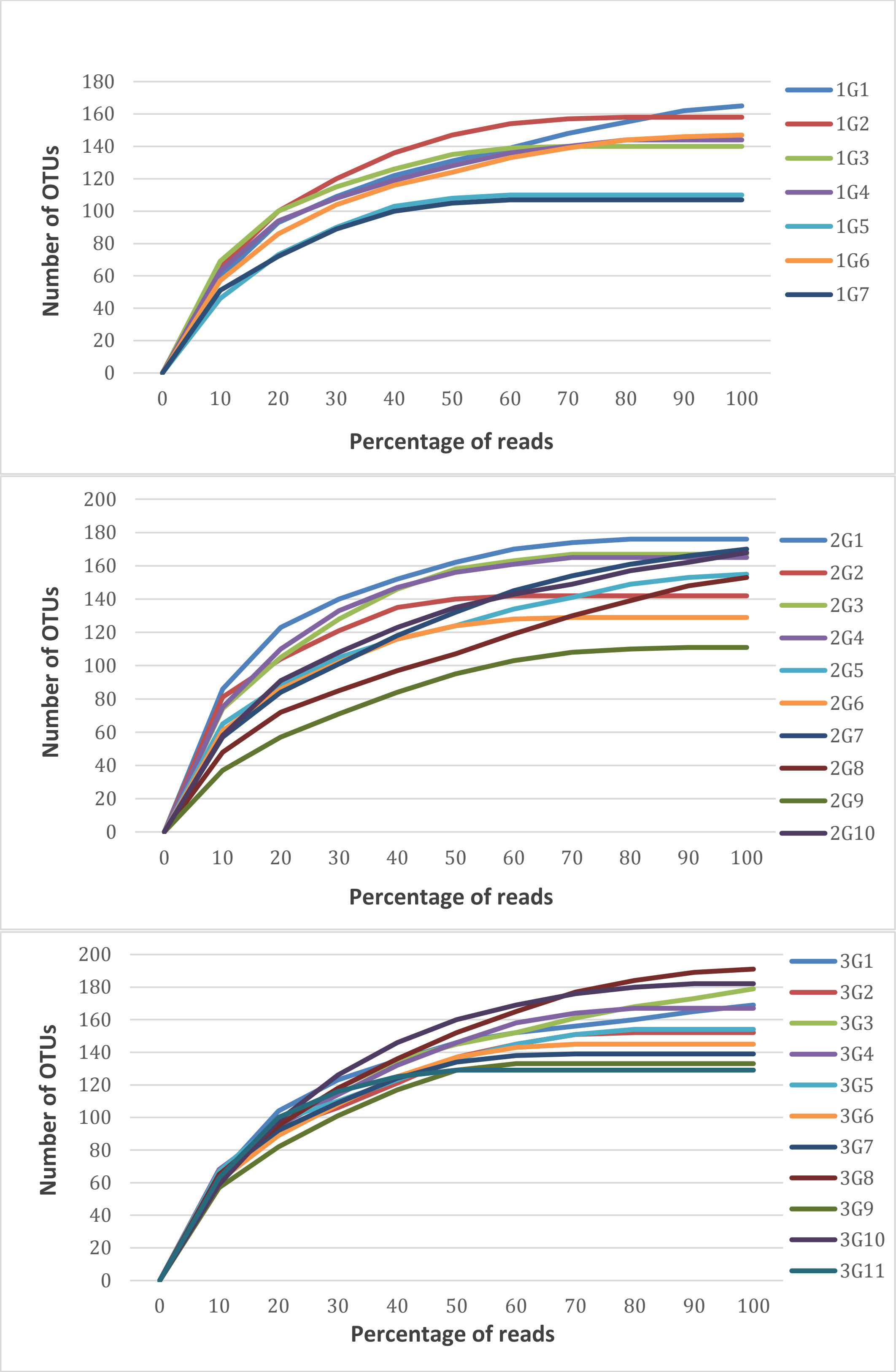

Fig. S1. Rarefaction curves of bacterial 16S rDNA gene sequences used to evaluate if further sequencing would likely detect additional taxa, indicated by a plateau.

Supplementary Figure S2.

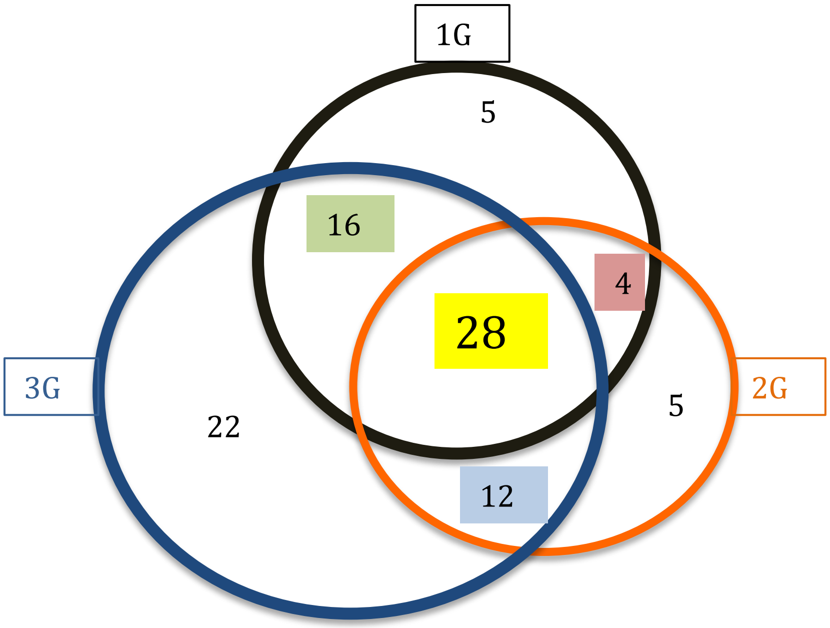

Fig. S2. Schematic representation of core microbiome at the species level. Black circle: number of species shared in all 1G mice. Orange circle: number of species shared in all 2G mice. Blue circle: number of species shared in all 3G mice. Intersections and numbers inscribed within refer to shared species at different levels of gravity.

Supplementary Figure S3.

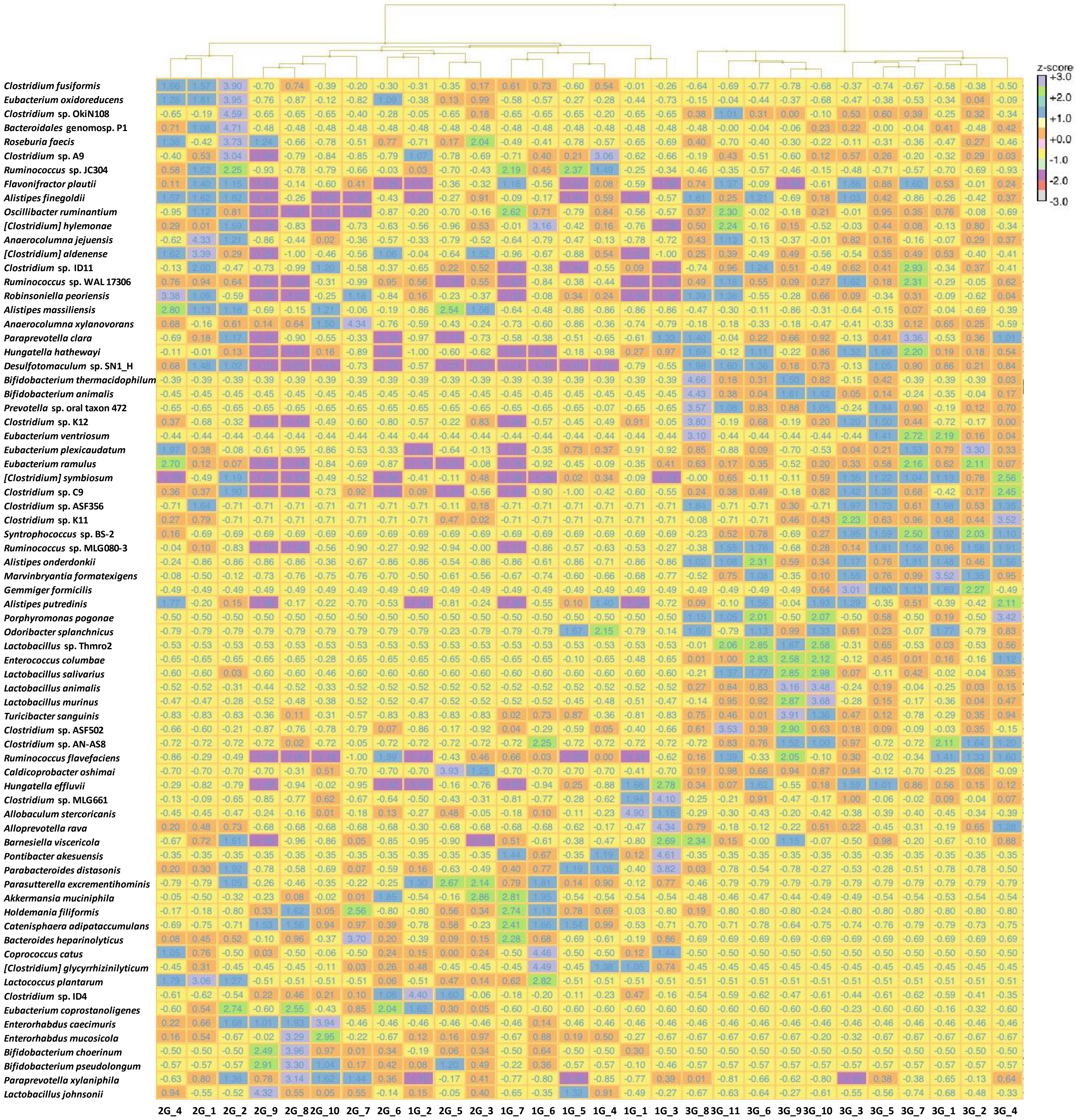

Fig. S3. Microbiota distribution at species level of taxon that contribute to sample variations (p>0.1). Heatmap shows square root-transformed read counts for the 73 taxa determined by similarity percentage analysis in each 1G, 2G and 3G samples. Numbers and colors represent the Z-scores, demonstrating all samples were represented by the median-centered Z-scores as the relative abundance levels. The upper dendrogram shows the clustering of samples based on Ward's hierarchical clustering method. For details, see Supplementary Dataset S1_g.

Supplementary Figure S4.

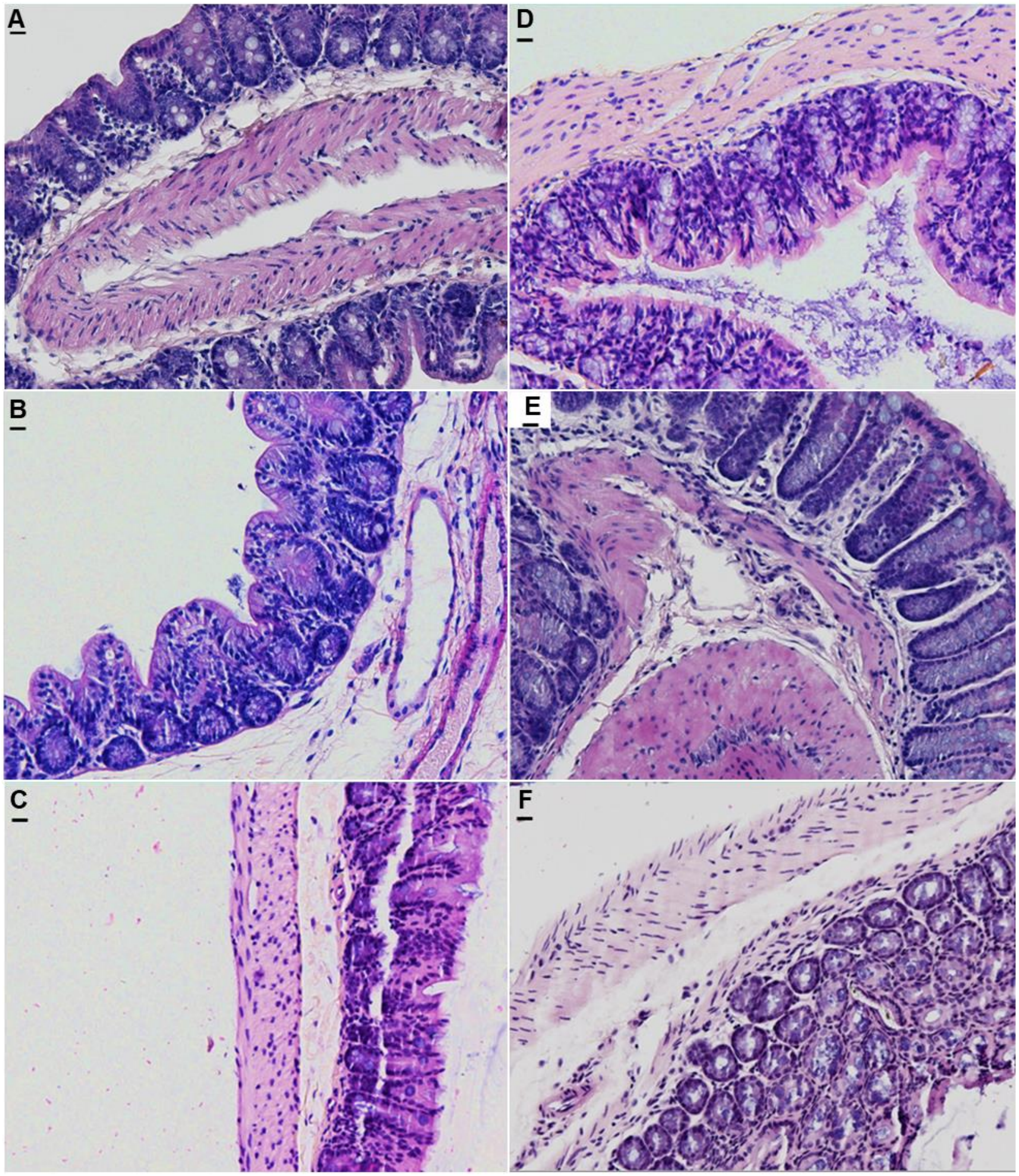

Fig. S4. HES stained caecal sections from control (1G) mice (A), 2G mice (B) and 3G mice (C) showing normal caecal mucosa without inflammation or epithelial change at 2 and 3G. HES stained colonic sections from 1G mice (D), 2G mice (E) and 3G mice (F) showing normal colonic mucosa without inflammation or epithelial change at 2 and 3G. Original magnification = 200x. Scale bar = 20 µm.

Supplementary Figure S5.

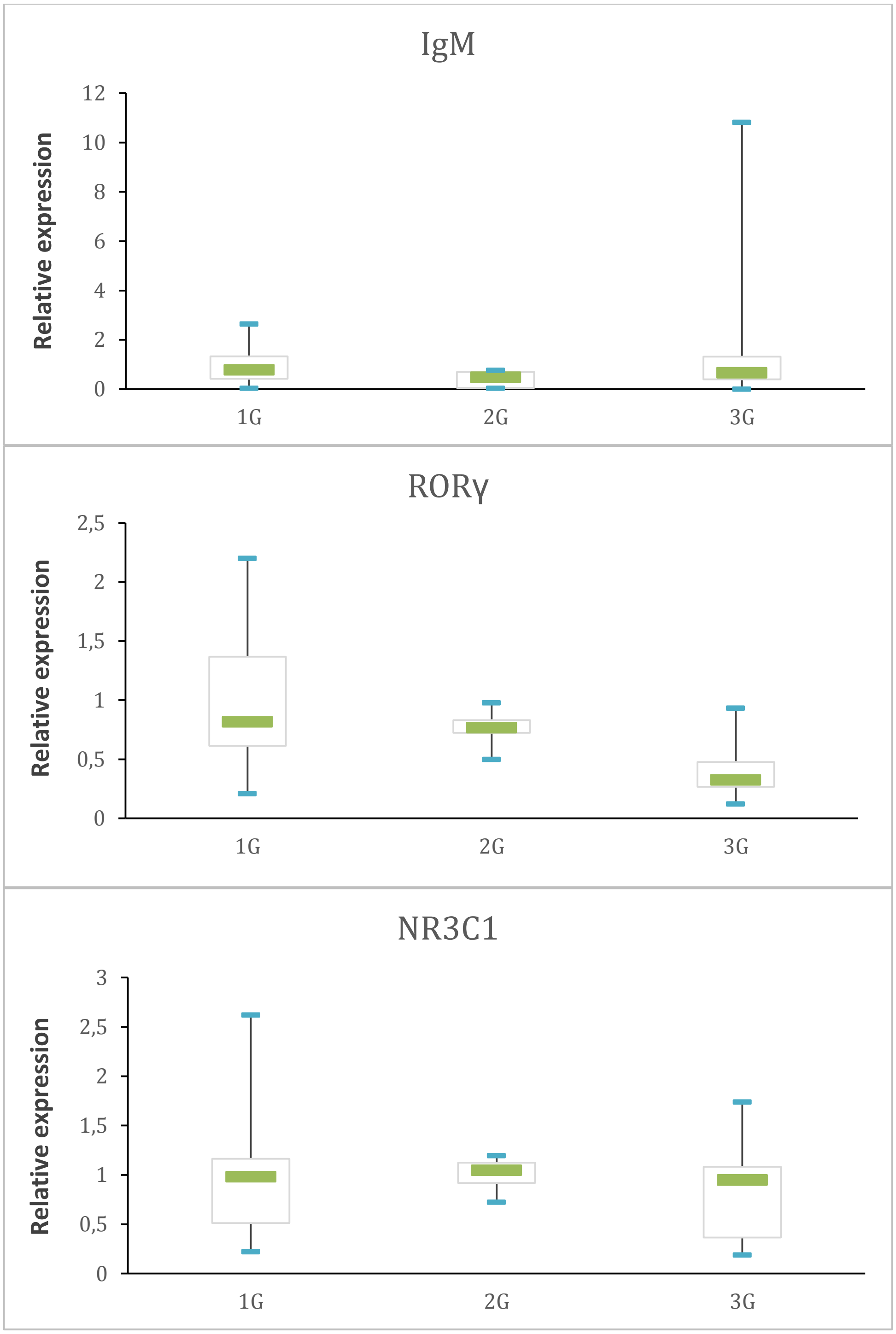

Fig. S5. Quantification of IgM, RORγ and NR3C1 mRNAs by qRT-PCR. mRNA levels were normalized to four housekeeping transcripts. Statistical analyses were done using the Mann-Whitney U test. The upper and lower ranges of the box represent the 75% and 25% quartiles, respectively. Error bars reflect standard error of the mean.

Supplementary Tables.

Table S1. 16S rDNA targeting primers used in this study for intracaecal bacterial load and specific relative abundance quantification by qPCR.

Bacterial group	Primer name	Primer sequence (5'–3')	Size (bp)	Reference
Universal *Bacteria*	1369F<br>1492R	CGGTGAATACGTTCCCGG<br>TACGGYTACCTTGTTACGACTT	123	Suzuki et al, 2000[1]<br>Lane 1991[2]
P. distasonis	Pdist1115F<br>Pdist1276R	CTTGCCACTAGTTACTAACA<br>CCCTGTCGCCAGGTG	162	This study
Paraprevotella clara	Pclara154F<br>Pclara251R	GGATGTGTTTGTCTTTCCGC<br>CTACCCATCGTYGCCTTGG	115	This study
Flavonifractor plautii	Fplautii170F<br>Fplautii423R	GGTCGCATGGCTCTGACT<br>TCATTTGTTTCGTCCCCGAC	272	This study
Parasutterella excrementihominis	Pexcr-817F<br>Pexcr-1001R	AAGTAAAATTCTCAGTAACGCAGC<br>GCTCTCATTACAAGAGCTTCC	184	This study

F, forward ; R, reverse

Table S2. Primers used in this study for quantification of immunological and stress markers in intestinal tissues.

Target	Primer sequence (5'–3')	Annealing T°C
Eef2	F : GTGGTGGACTGTGTGTCTGG<br>R : CGCTGGAAGGTCTGGTAGAG	58
Ppia	F : GTCTCCTTCGAGCTGTTTGC<br>R : GCGTGTAAAGTCACCACCCT	58
Eif3f	F : CATCAAGGCCTATGTCAGCA<br>R : AGGTCAACTCCAATGCGTTC	61
HRPT1	F : GTTGGATATGCCGACTA<br>R : GGCAACATCAACAGGACTCC	61
NR3C1	F : CAAGGGTCTGGAGAGGACAA<br>R : TACAGCTTCCACACGTCAGC	61
IgM	F : CTGGTGACCGAGAGGACCGT<br>R : GGAGGCAGTGGTCCACAGGT	61
Rorγ	QuantiTect Primer Assay (Qiagen, cat. no. QT00197722; product no. 249900)	61

F, forward ; R, reverse

Supplementary Database S1. Statistical analysis of intracaecal microbiomes comparing relative abundance using the nonparametric Mann-Whitney U test at the phylum level (Supplementary Dataset S1_a), at the class level (Supplementary Dataset S1_b), at the order level (Supplementary Dataset S1_c), at the family level (Supplementary Dataset S1_d), at the genus level genera (Supplementary Dataset S1_e) and at the species level (Supplementary Dataset S1_f). Megan data used for heatmap represented in Figure S3 (Supplementary Datasets S1_g).

Supplementary References.

1. Suzuki, M. T., Taylor, L. T. & DeLong, E. F. Quantitative analysis of small-subunit rRNA genes in mixed microbial populations via 5′-nuclease assays. *Appl. Environ. Microbiol.* **66,** 4605–4614 (2000).
2. Lane, D. 16S/23S rRNA sequencing. In 'Nucleic acid techniques in bacterial systematics'.(Eds E Stackebrandt, M Goodfellow) pp. 115–175. (1991).
